# Notoginsenoside R1 can inhibit the interaction between FGF1 and VEGFA to retard podocyte apoptosis

**DOI:** 10.1186/s12902-023-01402-6

**Published:** 2023-07-06

**Authors:** ChangYan Li, HuaChen Zhong, JingYuan Ma, Zhang Liang, Le Zhang, Tao Liu, WenXing Fan

**Affiliations:** 1 Department of Nephrology, the First Affiliated Hospital of Kunming Medical University, No.295, Xichang Road, Kunming, Yunnan Province 650032 China; 2 First Affiliated Hospital of Kunming Medical University, Kunming, Yunnan Province 650032 China; 3 Department of Science and Technology, Kunming Medical University, Kunming, Yunnan Province 650500 China; 4 Institute for Integrative Genome Biology, University of California Riverside, Riverside, CA 92521 USA; 5 Organ Transplantation Center, the First Affiliated Hospital of Kunming Medical University, Kunming, Yunnan Province 650032 China

**Keywords:** Notoginsenoside R1, VEGFA, FGF1, Diabetic nephropathy, Molecular docking

## Abstract

**Background:**

Diabetic nephropathy (DN) is a chronic condition resulting from microangiopathy in a high-glucose environment. The evaluation of vascular injury in DN has primarily focused on active molecules of VEGF, namely VEGFA and VEGF2(F2R). Notoginsenoside R1 (NGR1), a traditional anti-inflammatory medication, exhibits vascular activity. Therefore, identifying classical drugs with vascular inflammatory protection for the treatment of DN is a valuable pursuit.

**Methods:**

The “Limma” method was employed to analyze the glomerular transcriptome data, while the Spearman algorithm for Swiss target prediction was utilized to analyze the drug targets of NGR1. The molecular docking technique was employed to investigate the relationship between vascular active drug targets, and the COIP experiment was conducted to verify the interaction between fibroblast growth factor 1 (FGF1) and VEGFA in relation to NGR1 and drug targets.

**Results:**

According to the Swiss target prediction, the LEU32(b) site of the Vascular Endothelial Growth Factor A (VEGFA) protein, as well as the Lys112(a), SER116(a), and HIS102(b) sites of the Fibroblast Growth Factor 1 (FGF1) protein, are potential binding sites for NGR1 through hydrogen bonding. Additionally, the Co-immunoprecipitation (COIP) results suggest that VEGFA and FGF1 proteins can interact with each other, and NGR1 can impede this interaction. Furthermore, NGR1 can suppress the expression of VEGFA and FGF1 in a high-glucose environment, thereby decelerating podocyte apoptosis.

**Conclusion:**

The inhibition of the interaction between FGF1 and VEGFA by NGR1 has been observed to decelerate podocyte apoptosis.

**Supplementary Information:**

The online version contains supplementary material available at 10.1186/s12902-023-01402-6.

## Background

Diabetic nephropathy (DN) is a prominent etiological factor in end-stage renal disease (ESRD) [1]. It is a metabolic disorder that is influenced by numerous inflammatory factors, including high glucose, lipid metabolism disorder, oxidative stress, and advanced glycosylation products [2]. Despite being a crucial element in renal function, the mechanism of podocyte injury remains incompletely understood, resulting in the limited efficacy of traditional Chinese medicine in treating DN. Comprehending the pathogenesis of DN and harnessing the benefits of traditional Chinese medicine can broaden the scope of therapeutic alternatives and regimens for pharmaceutical interventions targeting DN.

Notoginsenoside R1 (NGR1) is a compound extracted from the rhizome of the Acanthaceae plant, panax notoginseng [3]. NGR1 has demonstrated therapeutic potential for various cardiovascular diseases, diabetes, and renal diseases, and exhibits pharmacological properties such as anti-oxidation, anti-inflammation, anti-angiogenesis, and anti-apoptosis [4]. However, the investigation of its underlying mechanism remains in its nascent stages.

Recent evidence indicates that Vascular endothelial growth factor (VEGFA) serves as a pro-angiogenic factor in endothelial cell proliferation, differentiation, migration, and permeability. Animal experimental studies have demonstrated that angiogenic factors can confer a protective effect on renal function in a mouse model of type 1 diabetes via angiopoietin-1 [5, 6]. Furthermore, clinical studies have established a close association between VEGFA and the progression of chronic kidney disease, and have revealed a synergistic effect between VEGFA and angiopoietin-1 [7, 8].VEGFA-based pharmaceuticals are a crucial therapeutic intervention for renal disease, supported by robust evidence and notable benefits. The present investigation employed the Swiss target prediction approach to anticipate the potential drug targets of NGR1, enriched the possible drug targets, and identified the fundamental genes of the primary enrichment pathway. Network pharmacological and molecular docking validation of core gene targets verified that NGR1 can mitigate podocyte damage by modulating the interaction between FGF1 and VEGFA.

## Materials and methods

### Data set selection

The present study sourced its data from a publicly available dataset offered by the gene expression synthesis database (http://www.ncbi.nlm.nih.gov/go/). Specifically, the experimental dataset utilized for this study was GSE30122 [9] of DN data, which comprised of 9 glomerular samples from DN patients and 26 normal controls. Additionally, GSE96804, which was previously described in literature, was composed of 41 glomerular samples from DN patients and 20 normal controls [10]. The identification of differentially expressed genes (DEGs) associated with DN was achieved by performing differential analysis on gene expression profiling data using the “limma” package, with a significance threshold of P < 0.05 and a LogFC value of 1, subsequent to standardization of the data.

### DEG’s GO and KEGG enrich the analysis

The Gene Ontology (GO) database employs a P-value threshold of less than 0.05 to screen proteomes for GO annotations. The objective of this study is to establish a standardized semantic vocabulary for diverse species and elucidate the biological significance of genes and proteins with P-values [11]. GO encompasses three primary categories, namely molecular function (MF), biological process (BP), and cellular component (CC), which facilitate the definition and description of gene functions. The modular clustering algorithm is utilized to cluster annotation items based on their degree of annotation, with a clustering value assigned to each cluster.

The significance and prominence of standards increase with higher scores, resulting in a higher ranking of the gene on the gene list. Furthermore, the KEGG database is a comprehensive resource that integrates genomic, chemical, and systemic functional information on genetic pathways across various species [12]. The R cluster analyzer package “org. hs. e.g., db” was utilized to uncover the biological functions and pathways associated with potential targets of autophagy genes. To summarize, the functional analysis was conducted utilizing R (version 4.2) and relied on the annotations from the Gene Ontology (GO) and Kyoto Encyclopedia of Genes and Genomes (KEGG) pathways. Additionally, statistical significance was determined by a P-value of less than 0.05.

### Molecular docking

To examine the regulatory impact of NGR1 on DN, the crucial gene and NGR1 were scrutinized and evaluated through molecular docking. The compounds’ structures were refined from PubChem (https://Pubchem.ncbi.nlm.nih.gov/) to reduce the binding energy. Furthermore, we acquired the 3D configuration of the human protein facilitated by core genes from the Protein Data Bank (https://www.rcsb.org/). Additionally, the water and small molecular ligands of the protein structure were eliminated utilizing AutoDockTools software [13]. Besides, the hydrogenation of the secondary systems facilitated the adjustment of the chemical structure of the active components within the active pocket range. The optimization of the docking results was carried out through the utilization of Pymol 2.5.1 software, and the most favorable outcomes were subsequently chosen.

### Genetic association analysis

Based on the gene expression of the target of DN treated by NGR1, the Spearman algorithm [14] was employed to examine the correlation between the marks based on the gene expression of the target of DN treated by NGR1. The screening threshold was set at P < 0.05. Spearman’s rank coefficient was utilized to determine the strength of correlation, with values ranging from 0.0 to 1.0. Specifically, a coefficient of 0.0 ≤ R ≤ 0.3 indicated no or very weak correlation, 0.3 < R ≤ 0.5 indicated weak correlation, 0.5 < R ≤ 0.7 indicated moderate correlation, 0.7 < R ≤ 0.9 indicated strong correlation, and 0.9 < R ≤ 1.0 indicated robust correlation. Furthermore, this manuscript pertains to the correlation among F2R, FGF1, ITGB5, and VEGFA genes individually.

### Detection of the activity of CC-K8

Human podocyte cells (HPCC) were seeded at a density of 5000 cells per well in 96-well plates and treated with HG, NGR1, or a combination of both. To assess cell viability, 10 µl of Dojindo Molecular Technologies (Kyushu, Japan) was added to each well, followed by incubation at 37 °C for 2 h. Absorbance was measured at 450 nM using a Bio-Rad (Hercules, CA) spectrophotometer. The experimental model utilized primary human kidney podocytes obtained from Shanghai Qincheng Biotechnology Co., Ltd. (product number: QC822) and cultured in a 37 °C incubator with 5% CO2. The podocyte injury model was established through the addition of anhydrous glucose to a culture medium consisting of 89% McCoy’s 5 A, 1% double-antibody, and 10% FBS, in a 30 mM high glucose environment for a duration of 24 h. Notoginsenoside R1 (NGR1) (R98% (HPLC)) was procured from Green Leaf Biotechnology Co., Ltd., No. B21099, Shanghai, and was prepared for use by dissolving the powder in DMSO and diluting it with the medium until the concentration of DMSO was less than 0.1%. Following HG treatment, NGR1 (1,3,10,30 µM) was administered to the cells once the cell density reached 60%, and was allowed to incubate for 24 h.

### Western blot

The methods employed in prior investigations are explicated in detail [15]. The specimens were derived from the same trial, and the gels/blots were processed concurrently. Additionally, the marker reagent was utilized to pre-stain the protein’s molecular weight, and the HPCC seed plate was positioned in a six-hole dish and treated with HG, NGR1, or both for 24 h to establish the model. Protein was extracted from RIPA tissue/cell lysate (Solebo, R0010, China) through repeated centrifugation at four °C for 5 min at 12000r/min. The protein concentration was determined using the BCA kit (BIYUNTIAN, NO.: P0010, China).

Following quantitative analysis, the protein was transferred onto a PVDF membrane (Millipore, no.: IPVH00010, Germany) via SDS-PAGE electrophoresis. Subsequently, a hypersensitive ECL Western HRP substrate (Zen-Bioscience Technology Co., No. 17,046, China) was utilized for development. The critical antibodies employed were VEGFA (No. 386,073, ZenbioN, China, 1:1000), FGF1 (No. 3843, ZenbioN, China, 1:1000), ITGB5 (No. R2473347, ZenbioN, China, 1:1000), Main Receptor (No. AF0263, Affin, China, 1:2000), and β-actin (No. ab82268, Abcam, UK, 1:1000).

### Quantitative. real-time polymerase chain reaction (qPCR)

In the present study, PBS (Phosphate Buffer Saline) was employed as a culture medium to facilitate the removal of cells. Subsequently, 1 ml of TRIzol reagent (Tiangen Biotech Co., Ltd., Beijing, China) was added and the resulting mixture was subjected to reverse transcription using the First Strand cDNA Synthesis Kit (Tiangen Biotech Co., Ltd., Beijing, China) per 10 cm2-grown cultured cells. The lysate was evenly distributed on the cell surface by laying it horizontally for a brief period, followed by cell lysis. Finally, the cells were removed using a pipette. The cells containing Lysate were transferred to a centrifuge tube and subjected to repeated pipetting to remove any visible precipitation. The resulting Lysate was allowed to stand at room temperature for 5 min. Subsequently, 1 ml of Trizol was added to each sample, followed by the addition of 0.2 ml of chloroform. The Lysate was vigorously shaken for 30 s and then incubated at room temperature for 3 min. The HEFF TM qPCR SYBR Green Master Mix Kit (Yepsen Biotech Co., Ltd., Shanghai, China) and a real-time fluorescence quantitative PCR system (MA-6000, Molarray) were used for Q-PCR analysis, and the 2-ΔΔCT method was utilized to determine the relative mRNA quantification. The expression of target mRNA was standardized to the housekeeping gene Glyceraldehyde 3-phosphate dehydrogenase (GAPDH) and exhibited as a fold change in comparison to the control group, primer sequences are itemized in Table 1.

**Table 1:** Sequence of Quantitative. Real-time polymerase chain reaction(qPCR)

Ollgo Name		Sequence	Length(bp)
GAPDH	Forward	ggc acc act act tca gag acc aag g	25
	Reverse	aca cga ggg cac aga aag caa tag	24
FGF1	Forward	aag aca ggc agg cag cac aat g	22
	Reverse	ggc aga aga gga aag gag cac ata g	25
VEGFA	Forward	gac agg gaa gag gag gag atg aga g	25
	Reverse	gaa gca ggt gag agt aag cga agg	24

### Co-immunoprecipitation assay

In order to establish a model of podocyte injury induced by hyperglycemia, cellular debris was isolated from samples collected after intervention with NGR1 through centrifugation at 13,000 X g for 10 min. Subsequently, r-protein A/G Magnetic (Nanjing ACE Biotechnology Co. Ltd, NO: AM002-01) and lysis/washing buffer (enhanced) were added to the magnetic beads, followed by incubation at room temperature for 10 min and adsorption of the sample through slight vortex mixing [16]. The magnetic beads were then incubated at room temperature for 10 min. Following the adsorption of magnetic beads, the supernatant was utilized as an eluent for protein extraction through the addition of a neutralization buffer measuring 5-10ml. To demonstrate protein-protein interaction, the WB method was employed and visualized using anti-VEGFA (Acidic, NO: ABIN6002755, Germany,1 mg/ml) and anti-FGF1 antibodies (Acidic, NO: ABIN1858859, Germany,5–20 µg/ml).

### Statistical methods

The experimental results were presented as the mean ± standard deviation. Statistical analysis was performed using SPSS 28.0 (Chicago, IL) with either a one-way analysis of variance or a Student’s t-test. A significance level of P < 0.05 was established and denoted by an asterisk.

## Results

### NGR1 is a potential target for DN

The involvement of vascular endothelial growth factor (VEGF) in the pathogenesis of DN is evidenced by its ability to modify the structure and function of endothelial cells [17], enhance vascular permeability, and induce stromal cell hypertrophy. The upregulation of VEGF expression is significantly linked to the onset of early proteinuria in DN. In diabetic rats, the renal expression of VEGF and its receptor mRNA is augmented, as demonstrated by previous investigations.

**Fig. 1:**
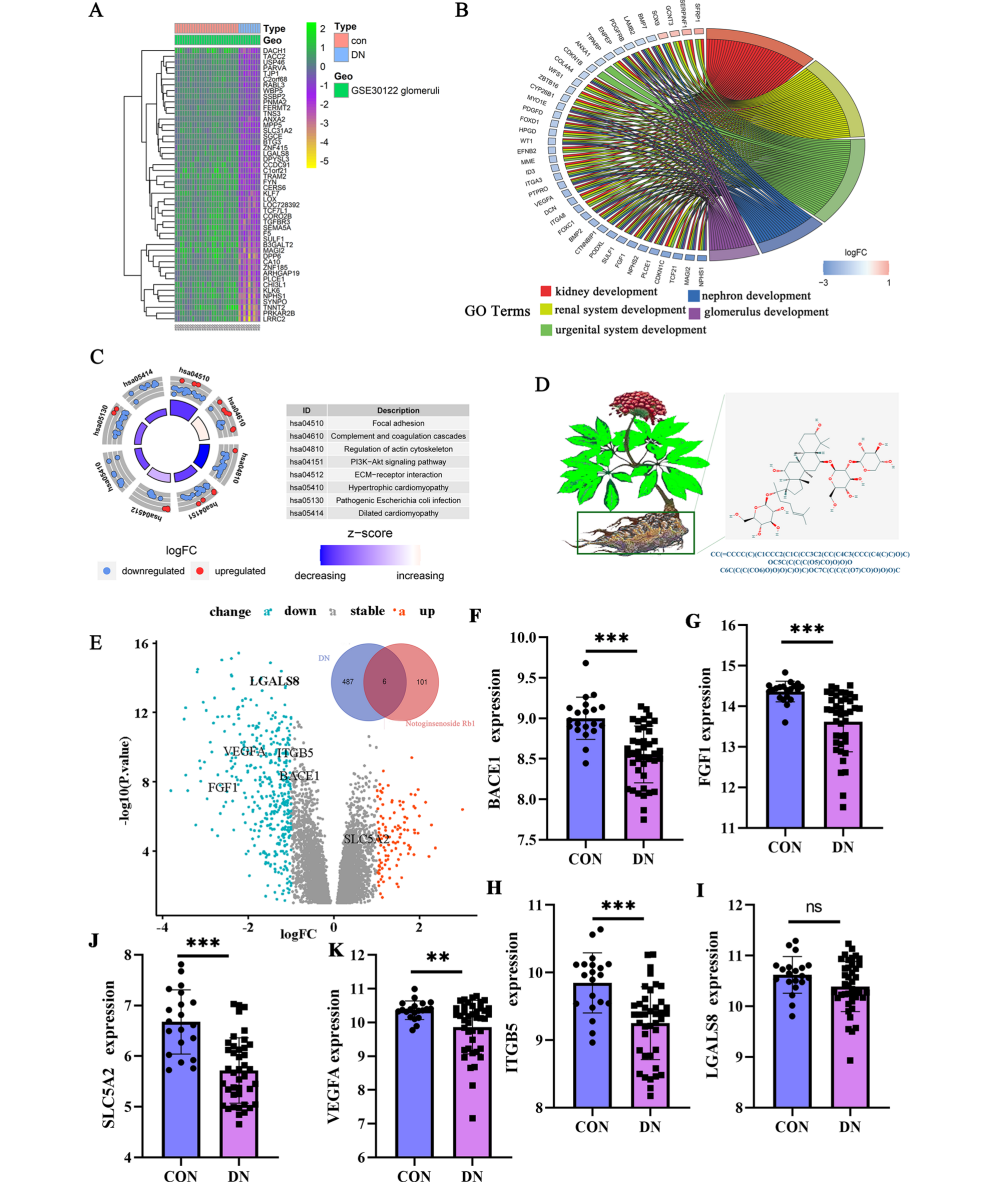
NGR1 has a therapeutic target of DN **A**: Differential analysis of glomerular samples of DN based on lemma packet; **B, C**: Differential gene function (GO) and signal pathway analysis (KEGG) of glomerular samples of DN; **D, E**: NGR1 and DN related differential gene Veen analysis; **F-K**:The changes of VEGFA, FGF1, ITGB5, SLC5A2, and BACE1 were verified based on GSE96804. *P < 0.05; **P < 0.01: ***P < 0.001

Syamentak Majumder posits that while this phenomenon may hold true for animal experiments, its applicability to human clinical studies remains a subject of controversy [18]. The present study also revealed this phenomenon, wherein differentially expressed genes that play a crucial role in renal uropoiesis and glomerular development were selected for experimentation (Fig. 1A, B, C). Furthermore, leveraging the canonical SMILES structure of NGR1 obtained from PubChem, we predicted its potential drug targets (Fig. 1D) and subsequently identified potential drug targets associated with glomerular injury in DN patients, such as VEGFA, FGF1, ITGB5, LGALS8, SLC5A2, and BACE1.

In this study utilized the GSE30122 database to investigate the gene expression levels of VEGFA, FGF1, and ITGB5 in DN patients, revealing a statistically significant decrease (P < 0.01) (Fig. 1E). To corroborate these findings, 41 DN glomerular samples and 20 control samples from the GSE96804 dataset were also examined, yielding consistent results. Notably, VEGFA exhibited a significant downregulation, which may have implications for the progression of DN. Furthermore, at the transcriptome level, significant alterations were observed in VEGFA, FGF1, ITGB5, LGALS8, SLC5A2, and BACE1 (Fig. 1F, G, H, I, J, K). This implies that NGR1 has the potential to modulate angiogenic functions, leading to amelioration of the pathological alterations associated with DN.

### Evaluation of the relationship between NGR1 potential target and DN function

VEGFA is primarily expressed by glomerular podocytes, as evidenced by previous research [19]. Furthermore, VEGFA mRNA has been detected in regions such as the renal tubules and collecting ducts, suggesting that VEGFA signaling is activated in podocytes but exerts its effects throughout the kidney [20]. VEGFA is capable of transmembrane diffusion and can bind to VEGFR-2, which is expressed on the surface of glomerular endothelial cells, thereby contributing to the normal development of the kidney [21].

**Fig. 2:**
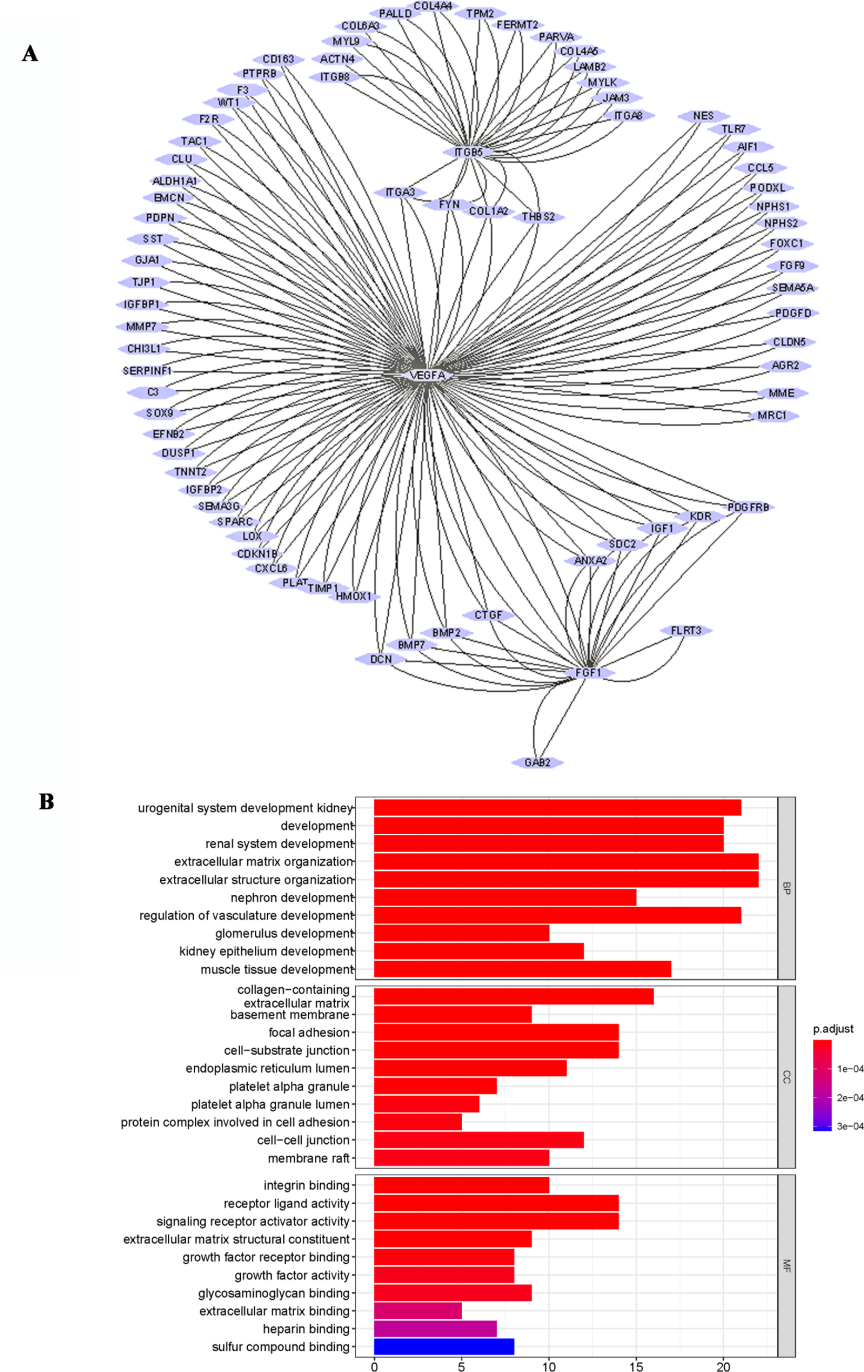
Progress of NGR1 in regulating DN through multiple pathways **A**: Protein interaction network of the pharmacodynamic target; **B**: Enrichment analysis of the expected target gene GO of NGR1 and DN.

The study revealed a significant decrease in the expression levels of VEGFA, FGF1, and ITGB5 genes in the DN group, although the precise role of VEGFA in this process remained unclear. To address this, the Spearman algorithm was employed to examine the interaction among VEGFA, FGF1, and ITGB5 (Fig. 2). The findings suggest a correlation between FGF1, ITGB5, and VEGFA, indicating the presence of mutual regulation among these genes.

**Fig. 3:**
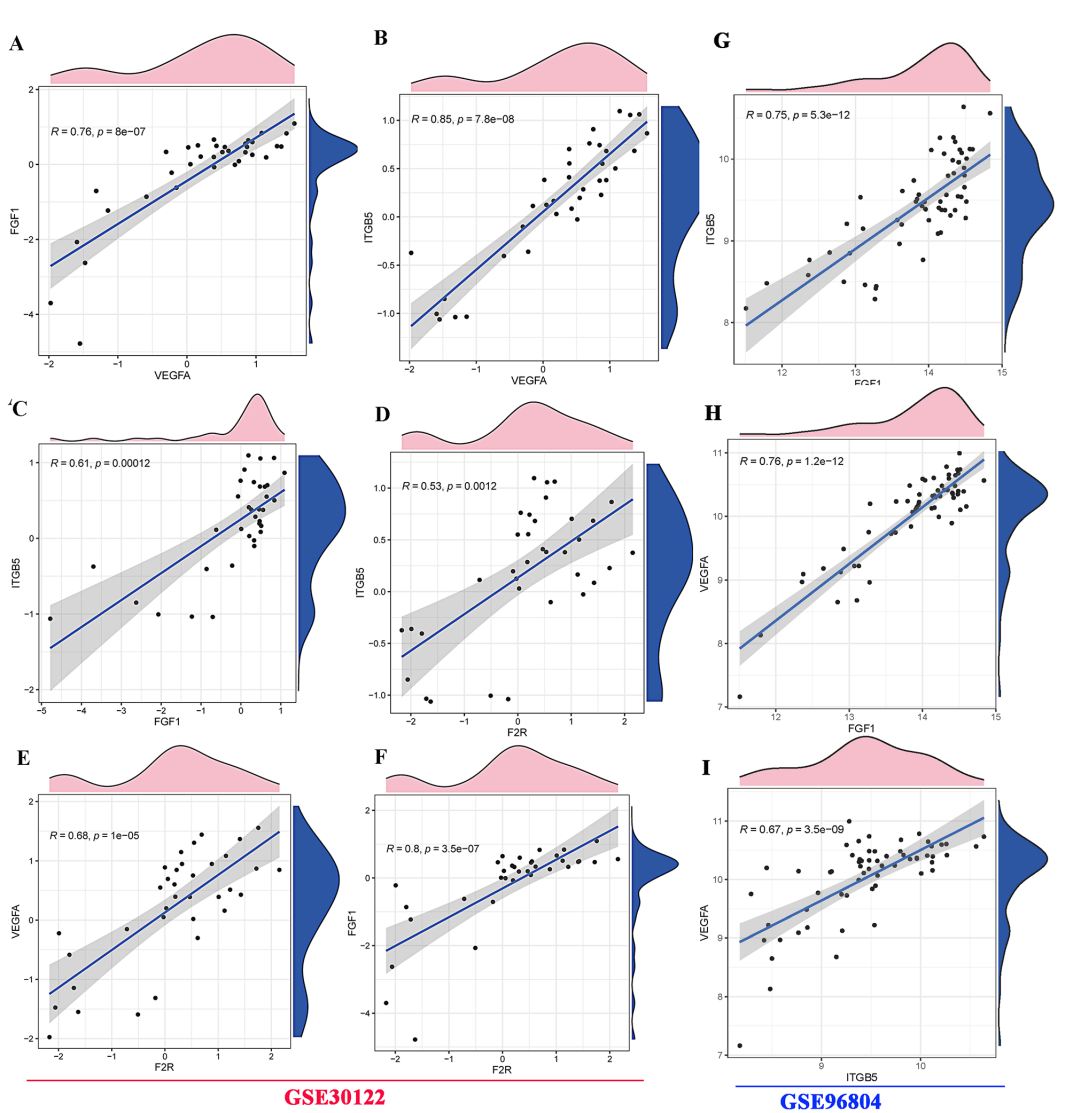
Gene correlation analysis by spearman algorithm. **A, B**: VEGFA, FGF1 and ITGB5 gene correlation analysis; **C, D**: ITGB5, FGF1 and F2R gene correlation analysis; **E, F**: F2R, VEGFA and FGF1 gene correlation analysis. **G-I**: GSE96804 was used to verify the interaction between the key interaction molecules VEGFA, FGF1 and ITGB5.

**Fig. 4:**
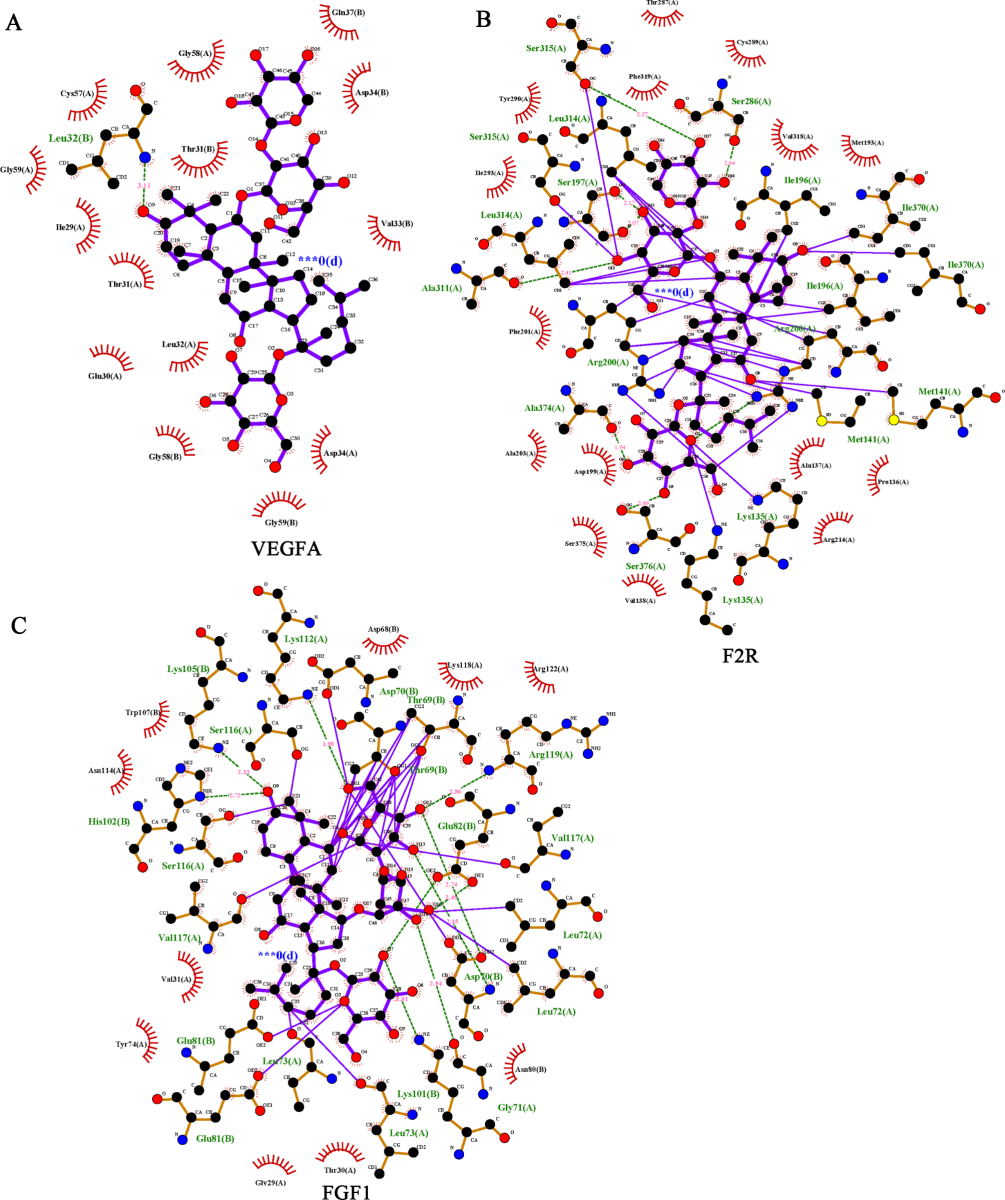
NGR1 can target the high glucose mediated by VEGFA, F2R and FGF1. **A**: NGR1 interacts with VEGFA, **B**: NGR1 interacts with FGF1; C: NGR1 interacts with F2R.

In order to assess the subunit interactions between NGR1 and the three active molecules, a molecular docking analysis was conducted using AutoDock Vina to simulate the potential interactions between NGR1 and the proteins. Additionally, Pymol 2.5 and Ligplot algorithms were utilized to analyze the binding modes, binding affinities, and critical interactions of each target’s maximum and minimum binding energies (Fig. 3). The results of the molecular docking analysis indicated that NGR1 exhibited an ability to interact with LEU32(b) of the VEGFA protein; Moreover, NGR1 exhibited significant hydrogen-bonding interactions with various F2R molecules, including SER315(a), Ser286(a), Ser376(a), Ala311(a), and Arg200(a). Additionally, the amino acid residues Lys112(a), SER116(a), and HIS102(b) of NGR1 demonstrated extensive hydrogen bond interactions with the FGF1 protein, as depicted in Fig. 4(A, B, C).

### NGR1 improves podocyte injury induced by high glucose

The podocyte is a significant constituent of typical renal function, serving as the primary cell responsible for the formation of filtration membranes. When damaged, it disrupts the overall glomerular filtration function [21]. The down-regulation of VEGFA expression occurs upon its destruction, indicating that a certain level of VEGFA is crucial in maintaining kidney homeostasis [22]. Additionally, it is the essential synthesis cell for vascular endothelial growth factor A (VEGFA) [23].

**Fig. 5:**
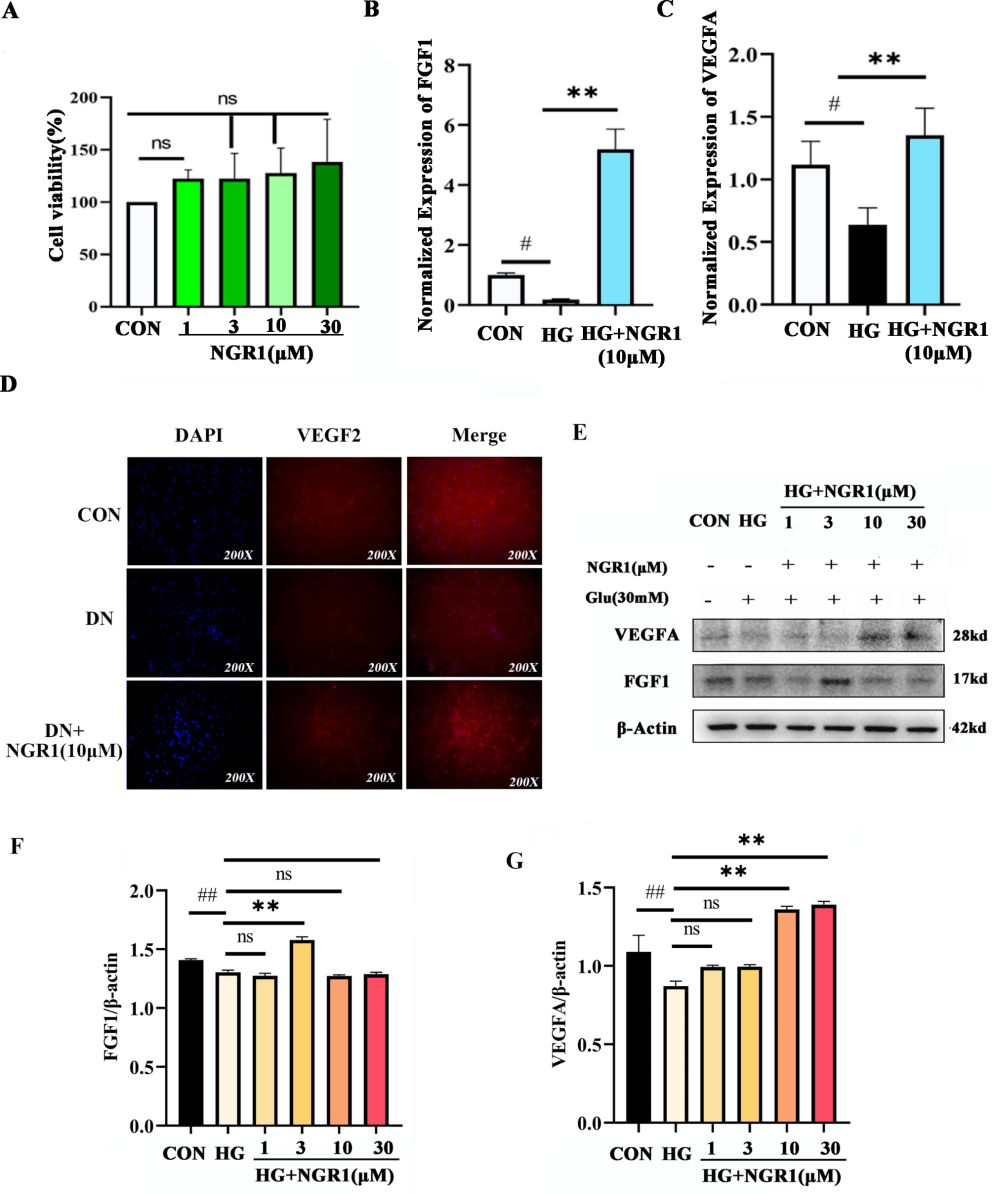
The samples derive from the same experiment and that gels/blots were processed in parallel. NGR1 regulates the protective effect of VEGFA and FGF1 on podocyte injury induced by high glucose. **A**: The cytotoxicity test of NGR1(1,3,10,30 µM) on cell viability (CCK8); **B**: The relative expression of FGF1; **C**: The relative expression of VEGFA; **D**: The expression of secretory protein VEGF2 was detected by immunofluorescence. * NGR1(10µM)vs HG, P < 0.05, ** NGR1 (10µM) vs HG, P < 0.01

This study utilized protein interaction analysis to demonstrate that FGF1 plays a role in the regulation of VEGFA. Specifically, the activation of FGF1 was observed to reduce blood glucose levels and delay podocyte damage resulting from high glucose exposure. Additionally, the potential for NGR1 to improve renal function and alleviate podocyte damage was investigated. To assess the cytotoxicity associated with podocyte injury in a high glucose environment, a model was constructed and evaluated using CCK-8 colorimetry. This was achieved by treating cells with or without glucose (30 µM) for 24 h to establish a high glucose environment/typical environment. In the course of the experiment, varying concentrations (1, 3, 10, 30 µM) of NGR1 and glucose (30 mM) were administered, and it was observed that they did not have a statistically significant impact on the viability of human renal podocytes (P < 0.05). The CCK-8 assay was conducted, and it was determined that NGR1 did not induce significant toxicity in human renal podocytes (Fig. 5A). Additionally, qPCR analysis revealed that the expressions of VEGFA and FGF1 were significantly reduced in a high glucose environment (Fig. 5B, C; P < 0.05). However, NGR1 was found to mitigate the high glucose-induced decrease of VEGFA and FGF1(Fig. 5D, E, F, G).

### NGR1 modulates VEGFA and FGF1’s protective effect on podocyte injury induced by high glucose

**Fig. 6:**
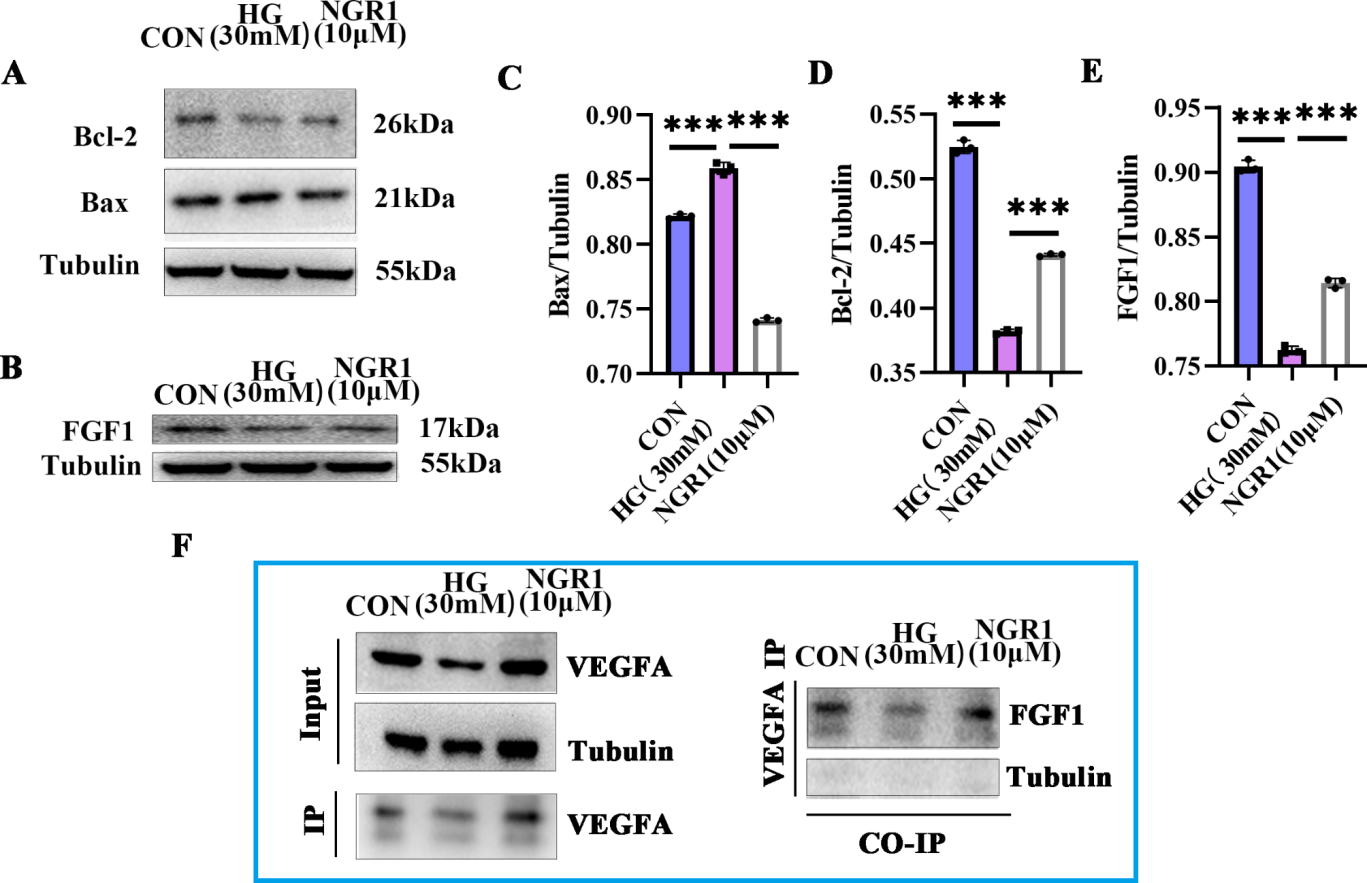
The samples derive from the same experiment and that gels/blots were processed in parallel. NGR1 ameliorates podocyte apoptosis through the interaction between FGF1 and VEGFA. **A, B**: Detection of FGF1, Bcl-2, Bax by WB; **C-E**: Statistical analysis of protein expression of FGF1, Bcl-2 and Bax under NGR1 intervention; **F**: CO-IP technique was used to detect the interaction of VEGFA and FGF1, as well as changes in the interaction of VEGFA and FGF1 after NGR1 intervention. *P < 0.05, **P < 0.01, ***P < 0.01

VEGFA, a vascular endothelial growth factor, has been shown to facilitate the formation of blood vessels and alterations in vascular permeability, while maintaining membrane integrity, which is essential for its functionality. Nevertheless, the precise function of VEGFA across the filtration membrane remains unclear. Our investigation has revealed that FGF1 can collaborate with VEGFA to provide protective effects against podocyte injury. Furthermore, fundamental experiments have substantiated the regulatory role of NGR1 in mitigating the mechanism of podocyte injury through the modulation of FGF1 and VEGFA. The administration of NGR1 (10 µM) to human glomerular podocytes under high-glucose conditions demonstrated a reduction in podocyte apoptosis induced by high-glucose (30 mM), with varying degrees of abnormal expression observed in both Bcl2 and Bax (Fig. 6A, C, D). The results of the study provide direct evidence of a strong interaction between VEGFA and FGF1 (Fig. 6B, E, F). These findings suggest that NGR1 may improve podocyte injury induced by high glucose, and the protective mechanism may be attributed to the inhibition of VEGFA and FGF1 expression in a high-glucose environment.

## Discussion

DN is a multifaceted chronic vascular inflammatory ailment that is influenced by various factors, including glucose and lipid metabolism, hemodynamic alterations, anomalous inflammatory responses, and cytokines such as transforming growth factor beta and vascular endothelial growth factor [24]. This condition is typified by inflammatory manifestations [25]. Prior research has indicated that several molecules associated with inflammatory pathways in diabetic nephropathy (DN) may present as innovative molecular targets for DN treatment, and the regulation of inflammatory responses holds considerable importance for DN prevention and treatment [26]. The glomerulus, which constitutes the capillary wall, experiences vascular degeneration that impairs the podocytes’ ability to sustain normal renal filtration.

This study aimed to investigate the involvement of DN-related genes in glomerular development and urinary tract formation at the transcriptome level. Our findings suggest that the therapeutic drug NGR1 may modulate the expression of VEGFA and FGF1 at the mRNA level by regulating crucial genes in the glomerulus and tubule, facilitating the interaction between VEGFA and FGF1, and preventing podocyte apoptosis, thus ameliorating podocyte injury.

VEGFA has been widely acknowledged as a promoter of angiogenesis, which enhances permeability and fosters the progression of diabetic nephropathy. As per the findings of Syamantak Majumder, a decline in the levels of VEGFA, a secretory protein whose reduction varies by cell type, is linked to human DN [27]. Furthermore, FGF1 is insulin-dependent and has the potential to lower glycated hemoglobin levels.

VEGFA has been identified as a significant contributor to the angiogenesis of DN. The present study has demonstrated a molecular interaction between VEGFA and FGF1, which serves a protective function in the progression of renal diseases. Additionally, the Spilman algorithm has been employed to elucidate the regulatory role of NGR1 drug targets through the analysis of the interaction between VEGFA, FGF1, and ITGB5. Notably, FGF1 has exhibited a positive correlation with VEGFA, thereby providing mechanistic insight into the synergistic action of these two factors.

Furthermore, AutoDock Vina conducted an investigation into the potential interactions between NGR1 and VEGFA, F2R, and FGF1, and subsequently analyzed the binding patterns, binding affinities, and key interactions of NGR1 with VEGFA and FGF1 utilizing Pymol 2.5 and Logplot. Ultimately, the findings indicate that NGR1 interacts with Leu32(B) of VEGFA, whereas it interacts with SER315(a), Ser286(a), Ser376(a), Ala311(a), and Arg200(a) of F2R. Additionally, NGR1 forms extensive hydrogen bonds with Lys112(a), SER116(a), His102(B), Kys101(B), Gly71(a), Asp70(b), and Arg119(a) of FGF1. The findings of this study indicate that NGR1 has the potential to mitigate kidney disease and exert regulatory effects on VEGFA, F2R, and FGF1. Subsequent investigation revealed that alterations in VEGFA and FGF1 were concomitant with changes in podocyte injury, leading to mRNA level modifications. Moreover, NGR1 was observed to facilitate the expression of both molecules. Notably, NGR1 was also found to promote protein binding between VEGFA and FGF1, thereby counteracting podocyte injury induced by high glucose.

The objective of this investigation was to examine the regulatory function of the potential therapeutic agent NGR1 in the progression of renal disorders. Our analysis revealed that NGR1 interacts with VEGFA and FGF1 at the molecular level, influencing their mRNA expression. Furthermore, NGR1 facilitates the protein binding between VEGFA and FGF1, potentially safeguarding podocytes from injury induced by high glucose levels. These findings suggest that NGR1 may confer a protective effect against kidney disease and regulate VEGFA, F2R, and FGF1. Our study provides novel insights into the role of NGR1 in renal disorders and may pave the way for the development of new therapeutic strategies.

## Electronic supplementary material

Below is the link to the electronic supplementary material.

Supplementary Material 1

## Data Availability

The gene screening section of the manuscript was analyzed using GSE30122 and GSE96804 samples from the NCBI GEO database, and all sequencing data are available free of charge from the GEO database (https://www.ncbi.nlm.nih.gov/GEO).
