# Supplementary material for: Notoginsenoside R1 can inhibit the interaction between FGF1 and VEGFA to retard podocyte apoptosis
Source: BMC Endocr Disord. 2023 Jul 6;23:140. doi: 10.1186/s12902-023-01402-6 (PMC10324173; doi:10.1186/s12902-023-01402-6)
Supplement: Supplementary file 1 — Supplementary Material 1 [file 12902_2023_1402_MOESM1_ESM.pdf]

WB strip exposure process

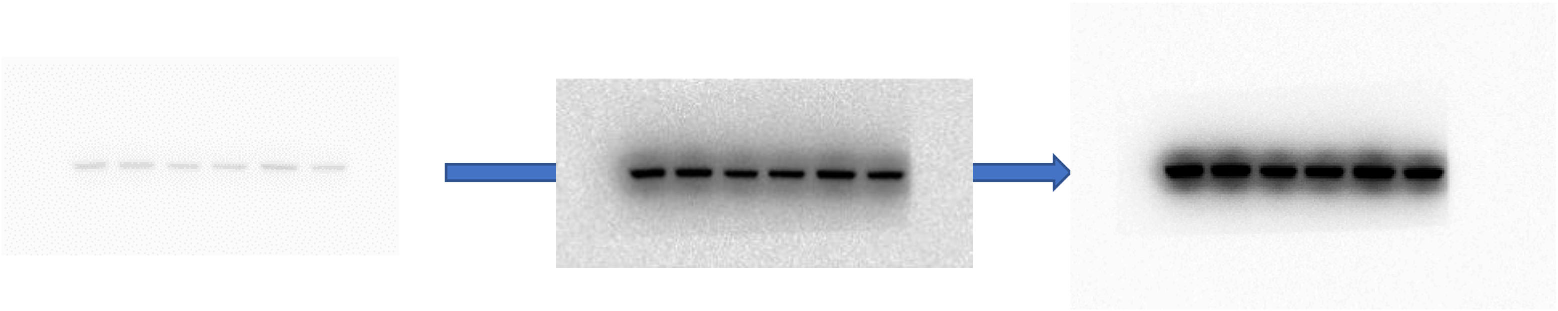

Figure 5E-β-Actin

WB strip exposure process

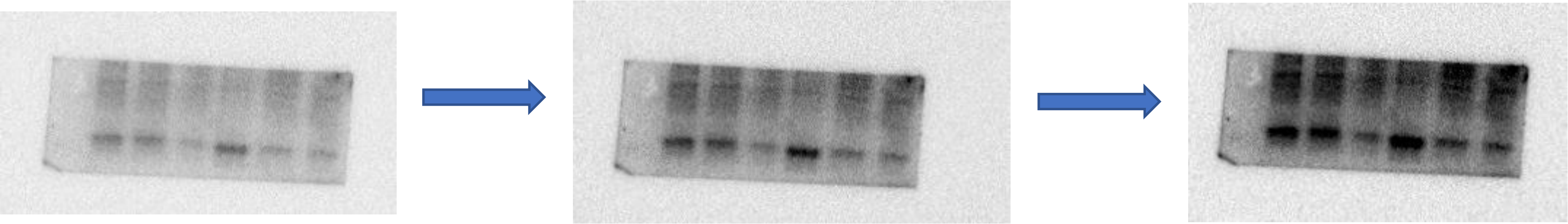

Figure 5E-FGF1

WB strip exposure process

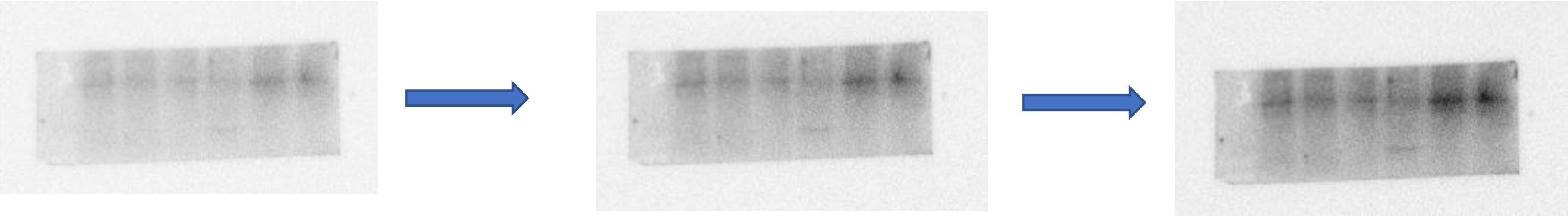

Figure 5E-VEGFA

WB strip exposure process

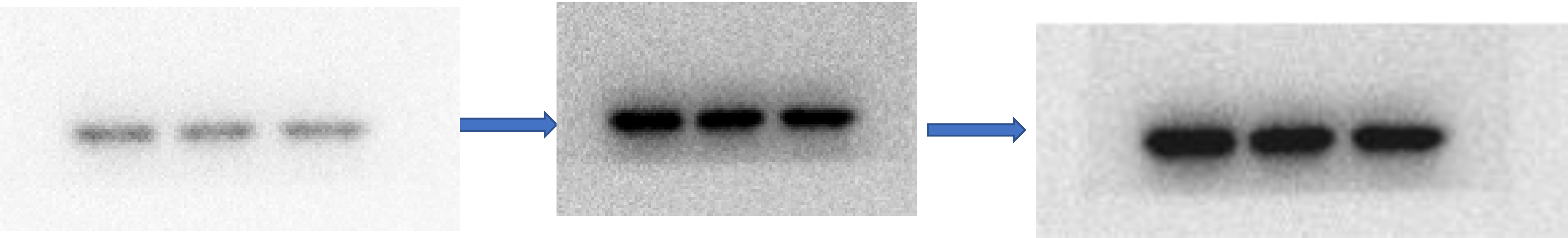

Figure 6A-Bax

WB strip exposure process

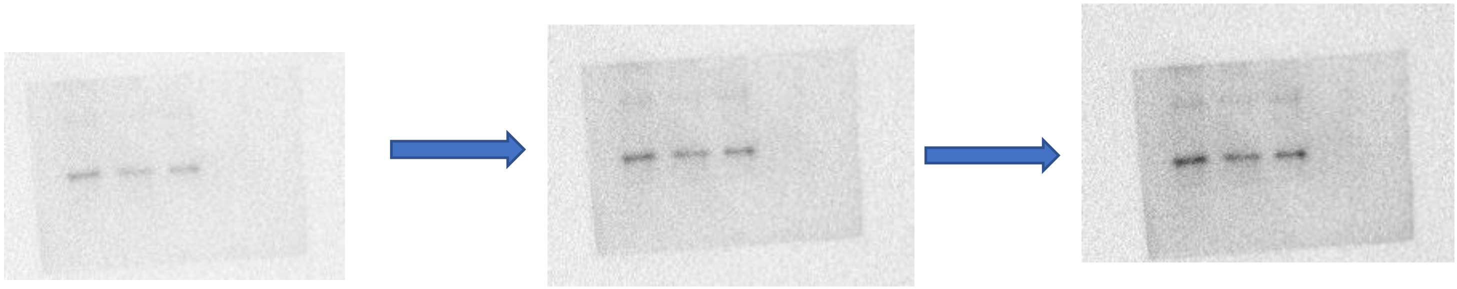

Figure 6A-Bcl2

WB strip exposure process

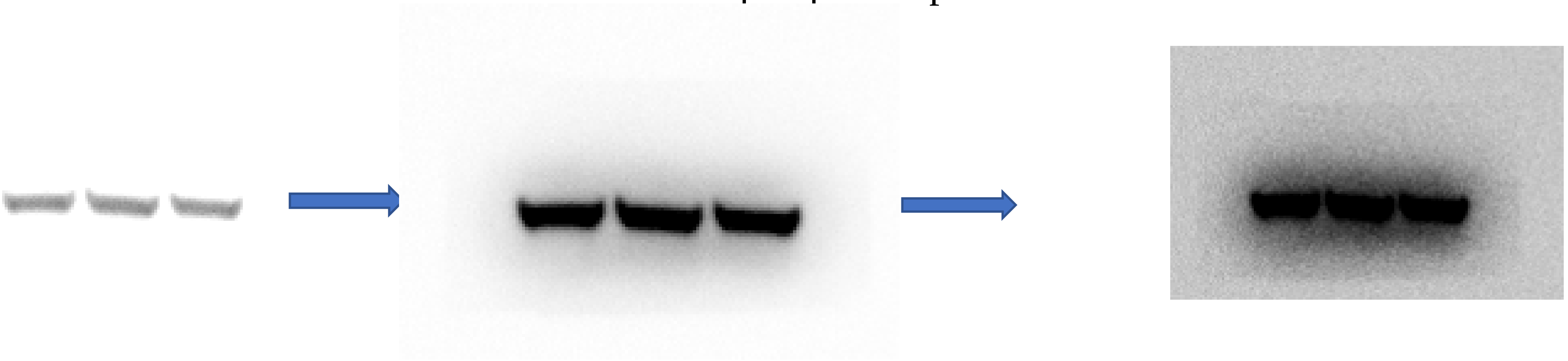

Figure 6A-Tubulin

WB strip exposure process

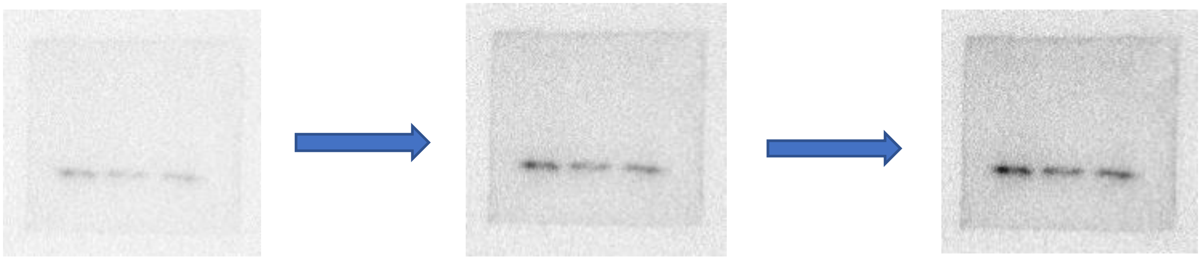

Figure6B-FGF1

WB strip exposure process

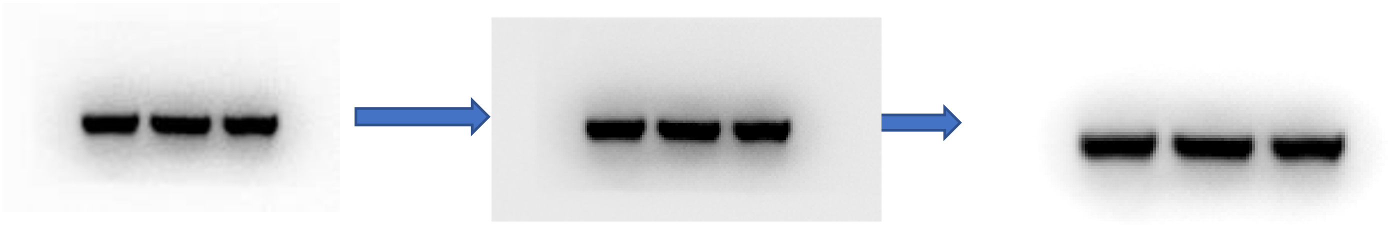

Figure6B-Tubulin

COIP strip exposure process

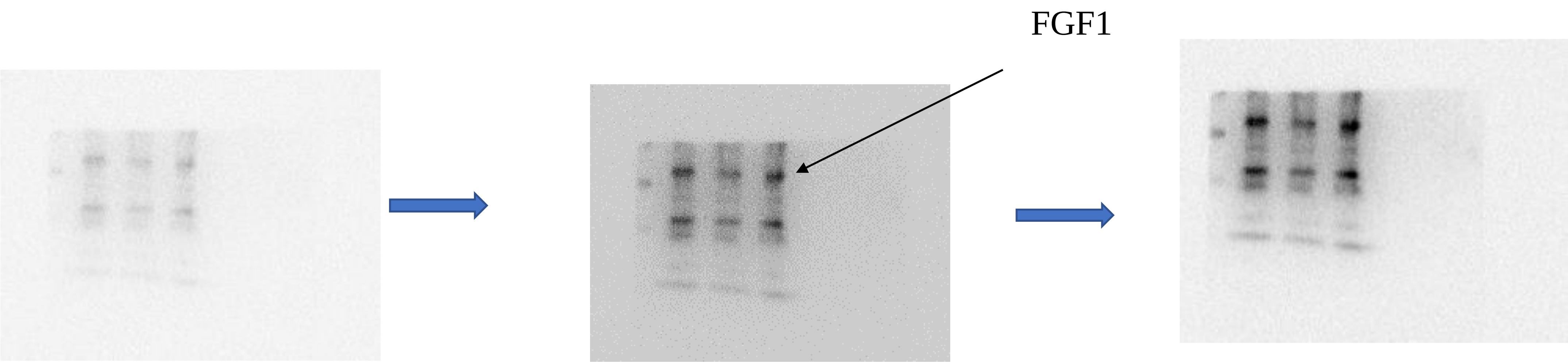

FGF1-COIP

COIP strip exposure process

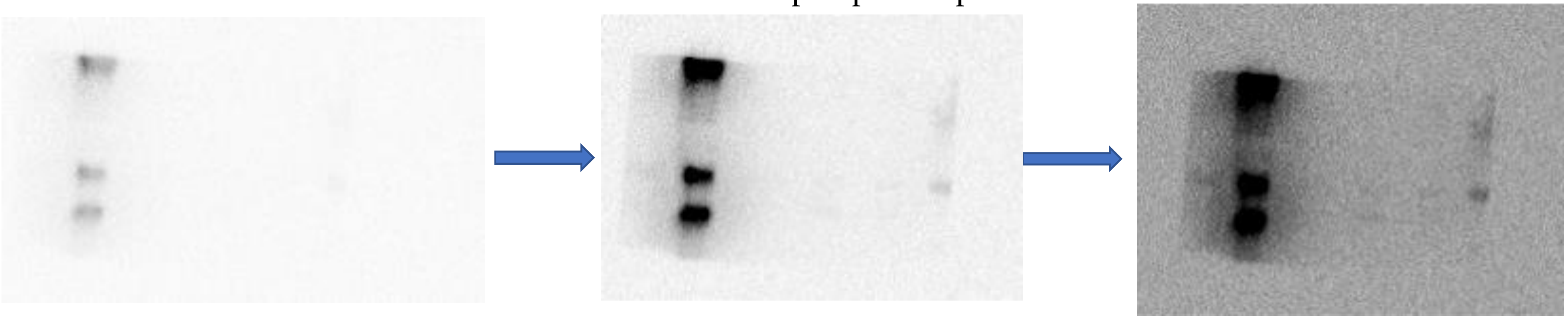

Tubulin-COIP

COIP strip exposure process

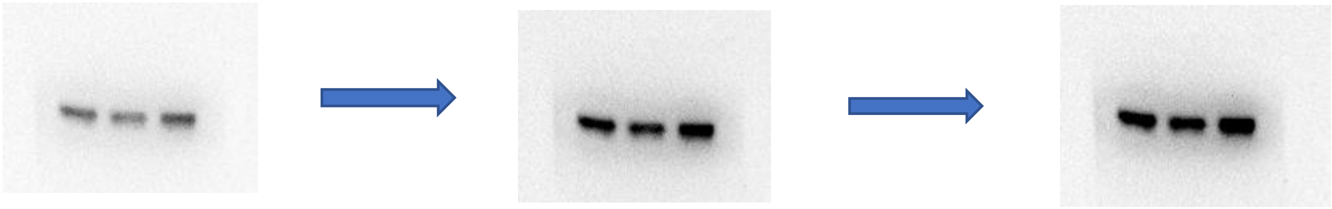

tubulin-Input

COIP strip exposure process

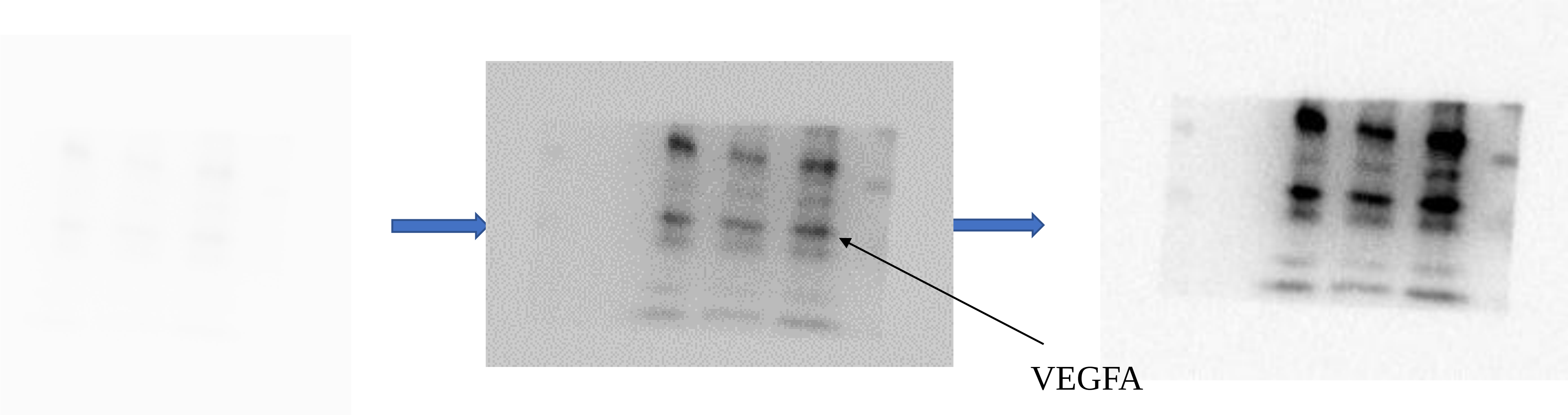

VEGFA-IP

COIP strip exposure process

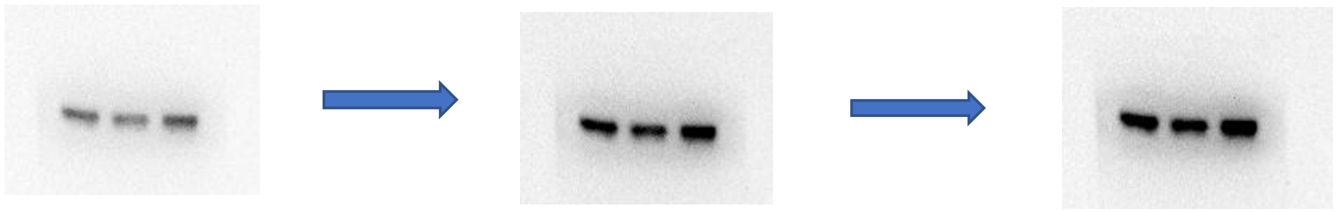

VEGFA-Input
